# Circular Permutation in Proteins

**DOI:** 10.1371/journal.pcbi.1002445

**Published:** 2012-03-29

**Authors:** Spencer Bliven, Andreas Prlić

**Affiliations:** 1 Bioinformatics Program, University of California, San Diego, La Jolla, California, United States of America; 2 San Diego Supercomputer Center, University of California San Diego, La Jolla, California, United States of America; University of Toronto, Canada

This is a “Topic Page” article for *PLoS Computational Biology*.

**Circular permutation** describes a type of relationship between proteins, whereby the proteins have a changed order of amino acids in their protein sequence, such that the sequence of the first portion of one protein (adjacent to the N-terminus) is related to that of the second portion of the other protein (near its C-terminus), and vice versa (see Figure 1). This is directly analogous to the mathematical notion of a cyclic permutation over the set of residues in a protein.

**Figure 1:**
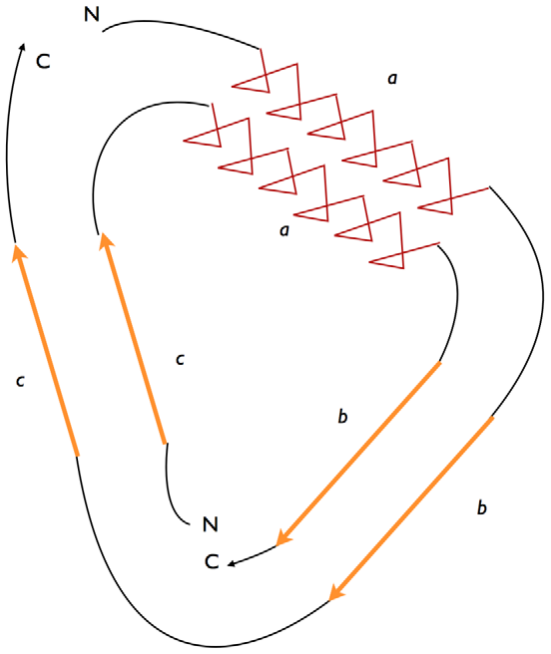
Schematic representation of a circular permutation in two proteins. The first protein (outer circle) has the sequence a-b-c. After the permutation the second protein (inner circle) has the sequence c-a-b. The letters N and C indicate the location of the amino- and carboxy-termini of the protein sequences and how their positions change relative to each other.

Circular permutation can be the result of evolutionary events, post-translational modifications, or artificially engineered mutations. The result is a protein structure with different connectivity, but overall similar three-dimensional (3D) shape. The homology between portions of the proteins can be established by observing similar sequences between N- and C-terminal portions of the two proteins, structural similarity, or other methods.

## History

**Figure 2:**
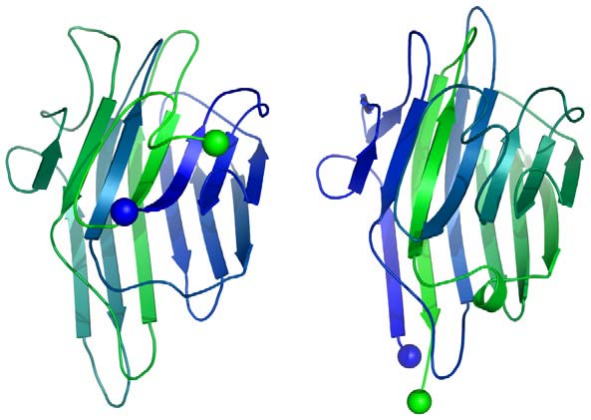
Two proteins that are related by a circular permutation. Concanavalin A (left), from the Protein Data Bank (PDB ), 3cna and peanut lectin (right), from PDB 2pel, which is homologous to favin. The termini of the proteins are highlighted by blue and green spheres, and the sequence of residues is indicated by the gradient from blue (N-terminus) to green (C-terminus). The 3D fold of the two proteins is highly similar; however, the N- and C- termini are located on different positions of the protein [1].

In 1979, Bruce Cunningham and his colleagues discovered the first instance of a circularly permuted protein in nature [1]. After determining the peptide sequence of the lectin protein favin, they noticed its similarity to a known protein—concanavalin A - except that the ends were circularly permuted (see Figure 2). Later work confirmed the circular permutation between the pair [2] and showed that concanavalin A is permuted post-translationally [3] through cleavage and an unusual protein ligation [4].

After the discovery of a natural circularly permuted protein, researchers looked for a way to emulate this process. In 1983, David Goldenberg and Thomas Creighton were able to create a circularly permuted version of a protein by chemically ligating the termini to create a cyclic protein, then introducing new termini elsewhere using trypsin [5]. In 1989, Karolin Luger and her colleagues introduced a genetic method for making circular permutations by carefully fragmenting and ligating DNA [6]. This method allowed for permutations to be introduced at arbitrary sites, and is still used today to design circularly permuted proteins in the lab.

Despite the early discovery of post-translational circular permutations and the suggestion of a possible genetic mechanism for evolving circular permutants, it was not until 1995 that the first circularly permuted pair of genes were discovered. Saposins are a class of proteins involved in sphingolipid catabolism and lipid antigen presentation in humans. Christopher Ponting and Robert Russell identified a circularly permuted version of a saposin inserted into plant aspartic proteinase, which they nicknamed swaposin [7]. Saposin and swaposin were the first known case of two natural genes related by a circular permutation.

Hundreds of examples of protein pairs related by a circular permutation were subsequently discovered in nature or produced in the laboratory. The Circular Permutation Database [8] contains 2,238 circularly permuted protein pairs with known structures, and many more are known without structures [9]. The CyBase database collects proteins that are cyclic, some of which are permuted variants of cyclic wild-type proteins [10]. SISYPHUS is a database that contains a collection of hand-curated manual alignments of proteins with non-trivial relationships, several of which have circular permutations [11].

## Evolution

There are two main models that are currently being used to explain the evolution of circularly permuted proteins: *permutation by duplication* and *fission and fusion*. The two models have compelling examples supporting them, but the relative contribution of each model in evolution is still under debate [12]. Other, less common, mechanisms have been proposed, such as “cut and paste” [13] or “exon shuffling.”

### Permutation by Duplication

**Figure 3:**
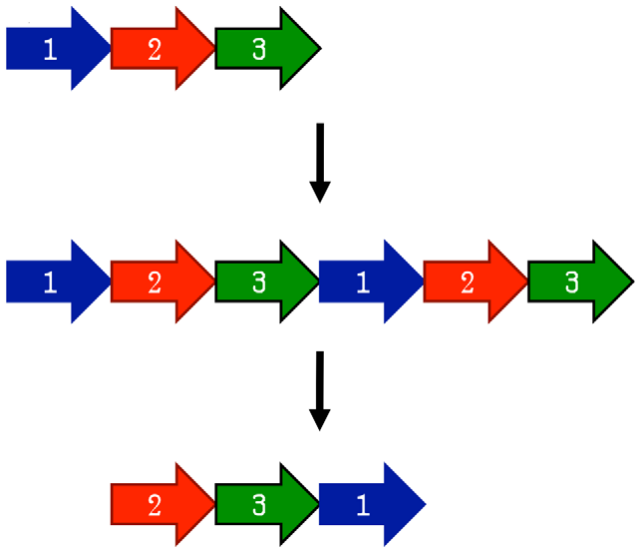
The permutation by duplication mechanism for producing a circular permutation. First, a gene is duplicated in place. Next, start and stop codons are introduced, resulting in a circularly permuted gene.

The earliest model proposed for the evolution of circular permutations is the permutation by duplication mechanism [1]. In this model, a precursor gene first undergoes a duplication and fusion to form a large tandem repeat. Next, start and stop codons are introduced at corresponding locations in the duplicated gene, removing redundant sections of the protein (see Figure 3).

One surprising prediction of the permutation by duplication mechanism is that intermediate permutations can occur. For instance, the duplicated version of the protein should still be functional, since otherwise evolution would quickly select against such proteins. Likewise, partially duplicated intermediates where only one terminus was truncated should be functional. Such intermediates have been extensively documented in protein families such as DNA methyltransferases [14].

#### Saposin and swaposin

**Figure 4:**
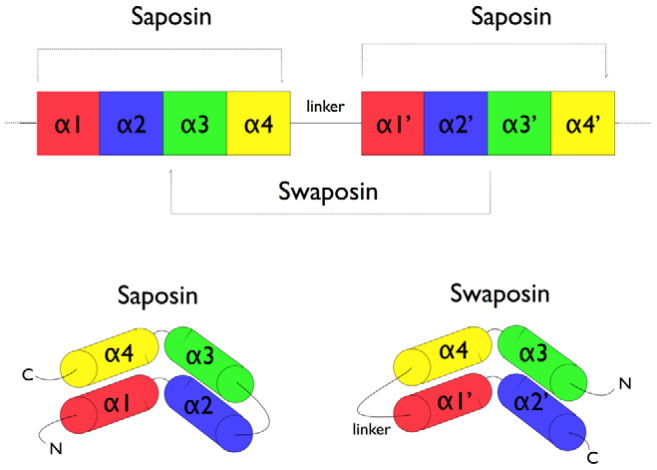
Suggested relationship between saposin and swaposin. They could have evolved from a similar gene [15]. Both consist of four alpha helices with the order of helices being permuted relative to each other.

An example for permutation by duplication is the relationship between saposin and swaposin. Saposins are highly conserved glycoproteins that consist of an approximately 80 amino acid residue long protein forming a four alpha helical structure. They have a nearly identical placement of cysteine residues and glycosylation sites. The cDNA sequence that codes for saposin is called prosaposin. It is a precursor for four cleavage products, the saposins A, B, C, and D. The four saposin domains most likely arose from two tandem duplications of an ancestral gene [16]. This repeat suggests a mechanism for the evolution of the relationship with the plant-specific insert (PSI) (see Figure 4). The PSI is a domain exclusively found in plants, consisting of approximately 100 residues and found in plant aspartic proteases [17]. It belongs to the saposin-like protein family (SAPLIP) and has the N- and C- termini “swapped”, such that the order of helices is 3-4-1-2 compared with saposin, thus leading to the name “swaposin” [7]. For a review on functional and structural features of saposin-like proteins, see Bruhn (2005) [18].

### Fission and Fusion

**Figure 5:**
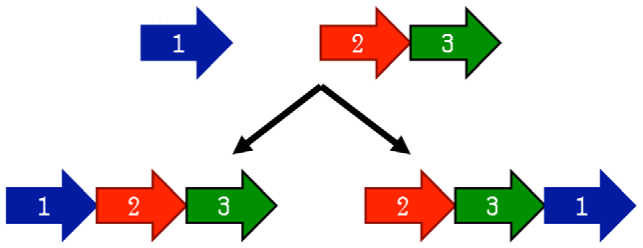
The fission and fusion mechanism of circular permutation. Two separate genes arise (potentially from the fission of a single gene). If the genes fuse together in different orders in two orthologues, a circular permutation occurs.

Another model for the evolution of circular permutations is the fission and fusion model. The process starts with two partial proteins. These may represent two independent polypeptides (such as two parts of a heterodimer), or may have originally been halves of a single protein that underwent a fissionfission event to become two polypeptides (see Figure 5).

The two proteins can later fuse together to form a single polypeptide. Regardless of which protein comes first, this fusion protein may show similar function. Thus, if a fusion between two proteins occurs twice in evolution (either between paralogues within the same species or between orthologues in different species) but in a different order, the resulting fusion proteins will be related by a circular permutation.

Evidence for a particular protein having evolved by a fission and fusion mechanism can be provided by observing the halves of the permutation as independent polypeptides in related species, or by demonstrating experimentally that the two halves can function as separate polypeptides [19].

#### Transhydrogenases

**Figure 6:**
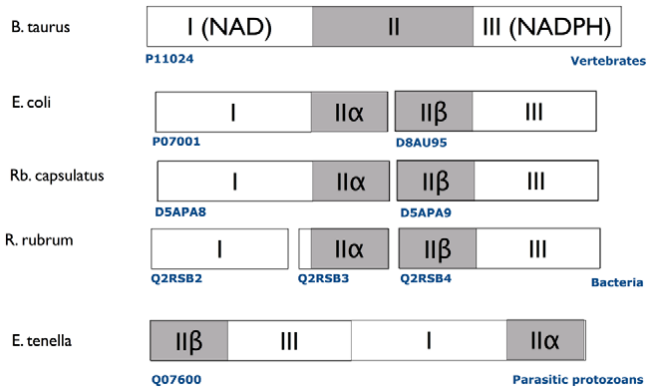
Transhydrogenases in various organisms can be found in three different domain arrangements. In cattle, the three domains are arranged sequentially. In the bacteria *E. coli*, *Rb. capsulatus*, and *R. rubrum*, the transhydrogenase consists of two or three subunits. Finally, transhydrogenase from the protist *E. tenella* consists of a single subunit that is circularly permuted relative to cattle transhydrogenase [20].

An example for the fission and fusion mechanism can be found in nicotinamide nucleotide transhydrogenases [20]. These are membrane-bound enzymes that catalyze the transfer of a hydride ion between NAD(H) and NADP(H) in a reaction that is coupled to transmembrane proton translocation. They consist of three major functional units (I, II, and III) that can be found in different arrangement in bacteria, protozoa, and higher eukaryotes (see Figure 6). Phylogenetic analysis suggests that the three groups of domain arrangements were acquired and fused independently [12].

### Other Processes that Can Lead to Circular Permutations

#### Post-translational modification

The two evolutionary models mentioned above describe ways in which genes may be circularly permuted, resulting in a circularly permuted mRNA after transcription. Proteins can also be circularly permuted via post-translational modification, without permuting the underlying gene. Circular permutations can happen spontaneously through auto-catalysis, as in the case of concanavalin A [4] (see Figure 2). Alternately, permutation may require restriction enzymes and ligases [5].

## The Role of Circular Permutations in Protein Engineering

Many proteins have their termini located close together in 3D space [21], [22]. Because of this, it is often possible to design circular permutations of proteins. Today, circular permutations are generated routinely in the lab using standard genetics techniques [6]. Although some permutation sites prevent the protein from folding correctly, many permutants have been created with nearly identical structure and function to the original protein.

The motivation for creating a circular permutant of a protein can vary. Scientists may want to improve some property of the protein, such as

- **Reduce** proteolytic **susceptibility**. The rate at which proteins are broken down can have a large impact on their activity in cells. Since termini are often accessible to proteases, designing a circularly permuted protein with less accessible termini can increase the lifespan of that protein in the cell [23].
- **Improve** catalytic activity. Circularly permuting a protein can sometimes increase the rate at which it catalyzes a chemical reaction, leading to more efficient proteins [24].
- **Alter substrate or** ligand binding. Circularly permuting a protein can result in the loss of substrate binding, but can occasionally lead to novel ligand binding activity or altered substrate specificity [25].
- **Improve** thermostability. Making proteins active over a wider range of temperatures and conditions can improve their utility [26].

Alternately, scientists may be interested in properties of the original protein, such as

- **Fold order**. Determining the order in which different parts of a protein fold is challenging due to the extremely fast time scales involved. Circularly permuted versions of proteins will often fold in a different order, providing information about the folding of the original protein [27]–[29].
- **Essential structural elements**. Artificial circularly permuted proteins can allow parts of a protein to be selectively deleted. This gives insight into which structural elements are essential or not [30].
- **Modify** quaternary structure. Circularly permuted proteins have been shown to take on different quaternary structure than wild-type proteins [31].
- **Find insertion sites for other proteins**. Inserting one protein as a domain into another protein can be useful. For instance, inserting calmodulin into green fluorescent protein (GFP) allowed researchers to measure the activity of calmodulin via the florescence of the split-GFP [32]. Regions of GFP that tolerate the introduction of circular permutation are more likely to accept the addition of another protein while retaining the function of both proteins.
- **Design of novel** biocatalysts **and biosensors**. Introducing circular permutations can be used to design proteins to catalyze specific chemical reactions [33], [24], or to detect the presence of certain molecules using proteins. For instance, the GFP-calmodulin fusion described above can be used to detect the level of calcium ions in a sample [32].

## Algorithmic Detection of Circular Permutations

Many sequence alignment and protein structure alignment algorithms have been developed assuming linear data representations and as such are not able to detect circular permutations between proteins. Two examples of frequently used methods that have problems correctly aligning proteins related by circular permutation are dynamic programming and many hidden Markov models. As an alternative to these, a number of algorithms are built on top of non-linear approaches and are able to detect topology-independent similarities, or employ modifications allowing them to circumvent the limitations of dynamic programming. Table 1 is a collection of such methods.

**Table 1:** Algorithms for comparing pairs of circularly permuted proteins.

Name	Type	Description	Author	Year	Availability	Reference
FBPLOT	Sequence	Draws dot plots of suboptimal sequence alignments.	Zuker	1991		[34]
Bachar et al.	Structure, topology independent	Uses geometric hashing for the topology independent comparison of proteins.	Bachar et al.	1993		[35]
Uliel at al.	Sequence	First suggestion of how a sequence comparison algorithm for the detection of circular permutations can work.	Uliel et al.	1999		[36]
SHEBA	Structure	Duplicates a sequence in the middle; uses SHEBA algorithm for structure alignment; determines new cut position after structure alignment.	Jung, Lee	2001		[37]
Multiprot	Structure, topology independent	Calculates a sequence order independent multiple protein structure alignment.	Shatsky	2004	Server, download	[38]
RASPODOM	Sequence	Modified Needleman and Wunsch sequence comparison algorithm	Weiner et al.	2005	Server	[39]
CPSARST	Structure	Describes protein structures as one-dimensional text strings by using a Ramachandran sequential transformation (RST) algorithm. Detects circular permutations through a duplication of the sequence representation and “double filter-and-refine” strategy.	Lo, Lyu	2008	Server	[40]
GANGSTA+	Structure	Works in two stages: Stage one identifies coarse alignments based on secondary structure elements. Stage two refines the alignment on residue level and extends into loop regions.	Schmidt-Goenner et al.	2009	Server, download	[41]
SANA	Structure	Detect initial aligned fragment pairs (AFPs). Build network of possible AFPs. Use random-mate algorithm to connect components to a graph.	Wang et al.	2010	Download	[42]
CE-CP	Structure	Built on top of the combinatorial extension algorithm. Duplicates atoms before alignment, truncates results after alignment.	Bliven et al.	2010	Server, download	[43]

The algorithms are classified according to the type of input they require. *Sequence*-based algorithms require only the sequence of two proteins in order to create an alignment. Sequence methods are generally fast and suitable for searching whole genomes for circularly permuted pairs of proteins. *Structure*-based methods require 3D structures of both proteins being considered. They are often slower than sequence-based methods, but are able to detect circular permutations between distantly related proteins with low sequence similarity. Some structural methods are *topology independent*, meaning that they are also able to detect more complex rearrangements than circular permutation.

**Further Reading**

- David Goodsell (2010) Concanavalin A and Circular Permutation. Research Collaboratory for Structural Biology (RCSB) Protein Data Bank (PDB) Molecule of the Month April 2010.
- Yu and Lutz (2011), for a review of the use of circular permutation in protein design [22].
- Weiner and Bornberg-Bauer (2006), for a review of evolutionary mechanisms for circular permutations [12].
- Cyclic permutation entry in Wikipedia, http://en.wikipedia.org/w/index.php?title=Cyclic_permutation

## Supporting Information

Text S1 Version history of the text file (XML); figures are also available in their original formats: Figure 1, Figure 2, Figure 3, Figure 4, Figure 5, Figure 6.
(XML)

Text S2 Peer reviews and response to reviews. Human-readable versions of the reviews and authors' responses are available as comments on this article.
(XML)
